# Health of Cities

**Published:** 1854-04

**Authors:** 


### HEALTH OF CITIES.

The health of large cities is becoming to be regarded more and more as a subject of highest importance. There is a great difference between them; in some cities one person out of every 25 dies annually, while in others there is only one death to every 50 persons. The following table exhibits several cities, in comparison, both European and American, showing in each how many inhabitants are lost by death every year:

| | | | | | |
|---|---|---|---|---|---|
| Portland | 1 in 62 | New York | 1 in 37 | Nice and Palermo | 1 in 31 |
| Philadelphia | 1 in 45 | St. Petersburg | 1 in 37 | Madrid | 1 in 29 |
| Glasgow | 1 in 44 | Charleston | 1 in 36 | Naples | 1 in 28 |
| Manchester | 1 in 44 | Baltimore | 1 in 35 | Brussels | 1 in 26 |
| Geneva | 1 in 43 | Leghorn | 1 in 35 | Rome | 1 in 25 |
| Boston | 1 in 41 | Berlin | 1 in 34 | | |
| London | 1 in 40 | Paris & Lyons | 1 in 32 | | |

The following is a comparison of six cities of the United S. for one week, ending Saturday, June 21, 1851:

| | Deaths. | Population. | Proportion. |
|---|---|---|---|
| Boston | 76 | 138,788 | 1 in 1,826 |
| New York | 330 | 517,849 | 1 in 1,569 |
| Philadelphia | 150 | 450,000 | 1 in 3.000 |
| Baltimore | 82 | 169,825 | 1 in 2,061 |
| Charleston | 17 | 48,014 | 1 in 3,584 |
| Savannah | 12 | 14,500 | 1 in 1,203 |

